# Precision nutrition in the context of bariatric surgery

**DOI:** 10.1007/s11154-023-09794-5

**Published:** 2023-03-17

**Authors:** Maite Aguas-Ayesa, Patricia Yárnoz-Esquíroz, Laura Olazarán, Javier Gómez-Ambrosi, Gema Frühbeck

**Affiliations:** 1 Department of Endocrinology and Nutrition, Clínica Universidad de Navarra, 31008 Pamplona, Spain; 2 Navarra Institute for Health Research, 31008 IdiSNA, Pamplona, Spain; 3 Centro de Investigación Biomédica en Red Fisiopatología de la Obesidad y Nutrición (CIBEROBN), 31008 Pamplona, Spain; 4 Metabolic Research Laboratory, Clínica Universidad de Navarra, 31008 Pamplona, Spain

**Keywords:** Bariatric surgery, Obesity, Deficiencies, Micronutrients, Protein, Weight loss, Precision nutrition

## Abstract

Bariatric surgery (BS) is the most effective long-term treatment for severe obesity. This review summarizes the main nutritional deficiencies before and after BS, as well as current dietary and supplementation recommendations to avoid them. Likewise, we have reviewed all those aspects that in recent years have been shown to be related to postoperative weight loss (WL) and its subsequent maintenance, such as hormonal changes, dietary patterns, changes in food preference, adherence to recommendations and follow-up, genetic factors and microbiota, among others. Despite all the knowledge, nutritional deficiencies and weight regain after BS are frequent. It is essential to continue studying in this field in order to establish more precise recommendations according to the individual characteristics of patients. It is also a major objective to understand more deeply the role of the factors involved in WL and its maintenance. This will allow the development of precision treatments and nutrition for patients with obesity, optimizing their benefit after BS.

## Introduction

Obesity has to be considered as a complex, multifactorial and chronic disease [1, 2]. Current treatment possibilities for obesity range from lifestyle modifications, including diet and exercise, to pharmacological, endoscopic and surgical treatment [3, 4]. Nowadays, bariatric surgery (BS) has become the most effective long term treatment for severe obesity and its comorbidities in carefully selected patients [5]. It is indicated in patients with a body mass index (BMI) above 40 kg/m^2^ or above 35 kg/m^2^ with obesity-related comorbidities such as type 2 diabetes, cardiovascular diseases, genitourinary, psychological and gastrointestinal alterations and obstructive sleep apnea, among others [6, 7]. Over the years, different BS procedures have been developed, being sleeve gastrectomy (SG) and Roux-en-Y gastric bypass (RYGB) the mostly used today [8, 9]. SG is a technique in which most part of the stomach is resected, creating a tubular gastric remnant based on the lesser curvature of the stomach [5], whereas RYGB is a technique that combines the resection of the upper part of the stomach creating a 30 ml pouch and a Roux-en-Y gastrojejunostomy [8].

Several mechanisms are involved in post-surgery weight loss (WL) achieved with these techniques that alter not only food intake, but also digestion and absorption of nutrients [10, 11]. In the case of RYGB, the gastric content does not pass through the first region of the small intestine, therefore, part of the nutrients are not absorbed. Nutrient digestion is also affected in SG as the removal of part of the stomach reduces intrinsic factor secretion and gastric acid production [12]. Furthermore, BS seems to alter the hormones involved in the regulation of hunger and satiety. After some bariatric procedures patients exhibit decreased fasting serum ghrelin levels and increased postprandial concentrations of glucagon-like peptide-1 (GLP-1), oxyntomodulin (OXM), and peptide YY (PPY) [13–18]. In addition, changes in the microbiota of the intervened patients as well as in the food preferences after BS have been observed [10].

Food intake is dramatically affected after BS, especially during the first year, because of the caloric restriction aimed at weight and fat loss. Furthermore, food intolerance may occur limiting the variety of foods consumed [19]. Thereby, an insufficient intake of some macronutrients, minerals and vitamins can take place, increasing the risk of deficient states with negative outcomes for the patients [20–22].

In addition, the high rates of pre-operative nutritional deficiencies in patients who are candidates for BS should be considered [23, 24] with their treatment before surgery being essential to prevent negative outcomes. Deficiency of vitamin A, vitamin B12, vitamin C, vitamin D, folic acid, calcium, iron, selenium and zinc are the most common before surgery. These deficiencies occur as a consequence of deficient intake due to inadequate nutritional habits, characterized by consumption of high energy-dense foods and low fruit and vegetable intake [22].

For all that, dietary recommendations for patients undergoing BS have been established [11, 25–28]. In addition, close monitoring of these patients is recommended to detect and treat potential deficiencies [11]. However, these recommendations are usually the same for different techniques and types of patients regardless of their specific individual characteristics and needs. This, together with the fact that follow-up of these patients is complex, makes nutritional deficiencies frequent [11, 12]. Moreover, weight regain is also common post-surgery. In this sense, periodic follow-up is essential for its prevention [12]. Likewise, it is necessary to know which dietary pattern could be more favorable for these patients to prevent weight regain [29]. For all these reasons, the development of precision nutrition approaches for patients undergoing BS is needed, that allow to obtain the maximum benefit of surgery preserving a correct nutritional status and the maintenance of WL preserving muscle mass. The present review analyzes the current body of knowledge about dietary and supplementation recommendations after BS, the main nutritional deficiencies before and after BS as well as other factors involved in WL and weight regain after BS.

## Pre-operative nutritional deficiencies

Nutritional deficiencies before BS are common. In fact, the rate of nutritional deficiencies is higher in people living with obesity (PLWO) candidates for BS than in the general population [23, 24]. Deficiency of vitamin B12, vitamin D, folic acid, calcium and iron are the most common before BS, although their prevalence varies according to age, sex, dietary patterns and other factors. These deficiencies are often multifactorial. On the one hand, the general intake of vitamins and minerals is altered by the low nutritional quality dietary patterns, rich in energy-dense foods but poor in fruits and vegetables, one of the main sources of vitamins and minerals [22, 30]. On the other hand, some deficiencies are due to conditions that occur specifically in PLWO. In the case of vitamin D, a decreased bioavailability of 25-hydroxyvitamin D because of sequestration by the adipose tissue is well established [31]. Some studies show that women with obesity have decreased expression of enzymes involved in 25-hydroxylation in adipose tissue compared to women with normal weight [32]. Moreover, there are other factors involved such as an unbalanced diet and lower sun exposure [31]. The absorption of iron may be decreased by the higher levels of hepcidin due to the subclinical inflammatory environment [30]. The identification of nutritional deficiencies prior to surgery and their treatment is essential not only for the patient to present an optimal nutritional status at the time of surgery but also to prevent the development of post-surgical deficiencies and their outcomes.

## Follow-up after BS: the key to success

### General recommendations

**Fig. 1:**
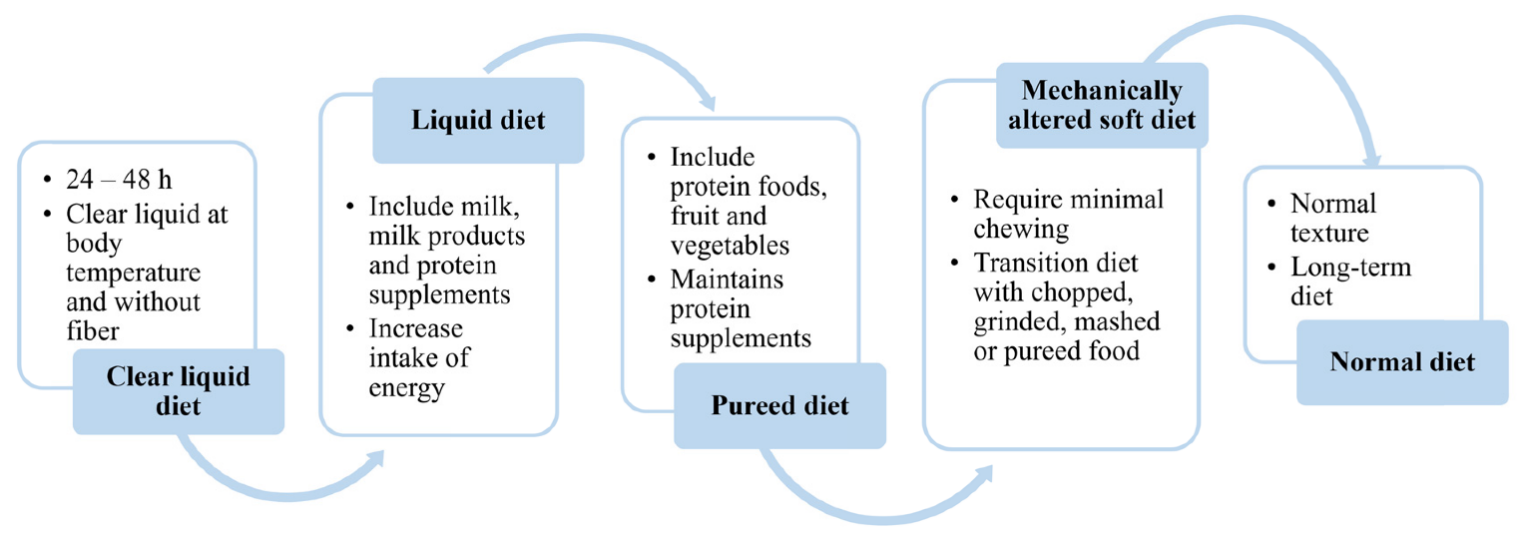
A progression diet is indicated after BS describing for each phase the amount, type and consistency of the foods included that are progressively increased

After BS nutrition must meet two main objectives: (i) to cover nutritional requirements in a context of energy restriction and (ii) to minimize the appearance of digestive complications such as intolerance, nausea, vomiting, reflux or dumping syndrome [25]. To achieve this, a progressive diet approach is indicated. The guidelines indicate some general recommendations, described in Table 1, but a unique protocol is lacking, and each center must adapt it to its own requirements [33]. Furthermore, it is recommended that the beginning and progression of the diet are guided by a registered dietitian, who should educate patients in a protocol-derived staged meal progression according to the type of surgery performed [11, 33]. Figure 1 shows the general characteristics of the progression diet. However, the duration of each phase, amount ingested, type of food and texture should be individualized according to the characteristics of the patient undergoing BS.

**Table 1:** General characteristics of an ideal diet after BS

**Resumption of oral intake**	A clear liquid meal regimen can usually be initiated 2 h postoperatively (27).
**Energy intake**	Start on average at 500 kcal with the liquid diet and increase progressively to 1,200 kcal per day in the solid intake phase.
**Protein requirement**	1.0-1.5 g/kg of ideal weight or 60–80 g/d (11,33).
**Hydration**	Intake of more than 1.5 L/d (11). Drink slowly and at least 30 min after meals to ensure a good digestion and prevent early satiety or other gastrointestinal symptoms (11). Clear liquids such as water and tea, avoiding the intake of liquids with sugar, caffeine, carbonation and alcohol are indicated.
**Other recommendations**	The dietary regimen should be structured in small and frequent meals during the day, patients should chew small bites of food thoroughly before swallowing (25). Include at least 5 daily servings of fresh fruits and vegetables to ensure a correct fiber intake (11,27).

### Nutritional requirements and deficiencies after BS

**Fig. 2:**
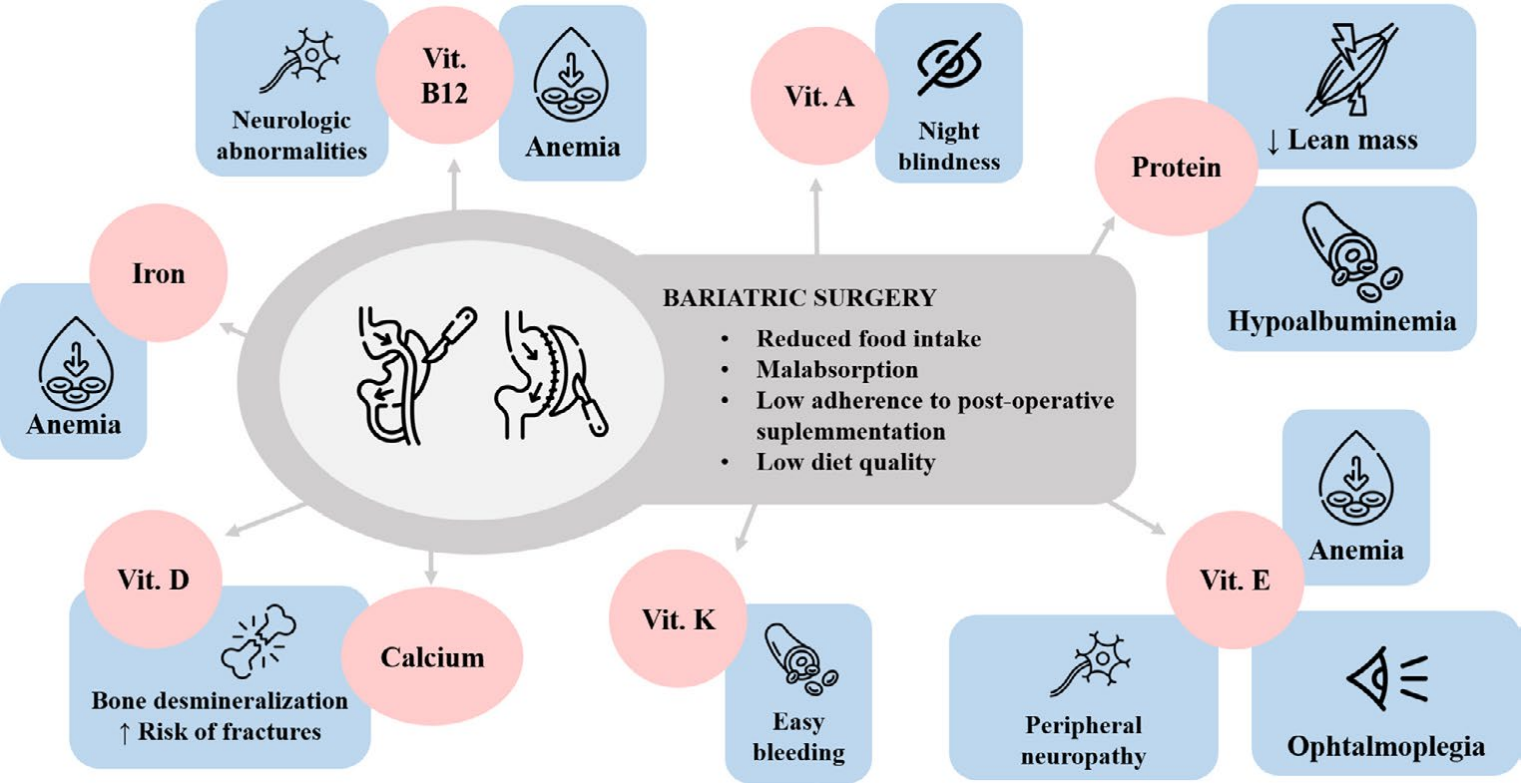
Common nutritional deficiencies after BS and their accompanying alterations Vit: vitamin

After BS the gastrointestinal tract undergoes changes in its anatomy and physiology that make patients susceptible to developing nutritional deficiencies, of both macro- and micronutrients, as well as proteins, which can lead to deficient states and diseases such as anemia, osteoporosis or protein malnutrition, among others (Fig. 2) [12]. The prevalence of nutritional deficiencies varies according to (i) surgical technique used, (ii) specific guidelines, (iii) preoperative micronutrient concentrations and (iv) the adherence of patients to dietary recommendations and supplementation. Regarding macronutrients, the most common deficiency after BS is protein deficiency. In contrast, micronutrient deficiency affects a large number of vitamins and minerals.

#### **Protein deficiency**

After BS protein intake is conditioned due to the low food intake, protein food intolerance and reduced digestibility, as well as protein malabsorption [34]. Protein deficiency is one of the most severe complications after BS. It is diagnosed when serum albumin levels are < 35 g/L and some of the symptoms include oedema, multi-organ failure and even death. Its prevalence differs according to the type of surgery, being more prevalent in surgeries with a malabsorptive component than in those with a restrictive one [20].

A further essential aspect after BS is to minimize the loss of lean mass, especially during the first months after BS when the energy intake is very low, and WL occurs very fast. Furthermore, protein intake amount exacts an important role in maintaining the basal metabolic rate [35]. In this context, some studies have demonstrated that a protein intake of 60 g/day is associated with a better lean mass retention after BS [36, 37]. Current recommendations of protein requirements indicate a protein intake between 60 and 80 g/day or up to 1.5 g/kg ideal body weight per day [11]. However, these protein intake recommendations are not based on studies specifically designed to assess protein requirement in this population [21]. In 2019 the update of the Clinical Practice Guidelines for the Perioperative Nutrition, Metabolic, and Nonsurgical Support of Patients Undergoing Bariatric Procedures indicated that the protein requirements should be individualized, assessed, and guided by a registered dietitian, regarding gender, age, and weight [11]. In some situations, with increased protein requirements, up to 2.1 g/kg of ideal body weight per day may be necessary [11]. Likewise, in the same year, European Society for Clinical Nutrition and Metabolism (ESPEN) studied the protein requirements of BS patients using the validated method of nitrogen balance, showing that protein requirements of PLWO were higher than those of people with normal weight. Furthermore, protein requirements of patients decreased in the third month after BS and increased in month 12. These changes may underlie metabolic changes occurring after BS although more studies are needed [21].

Achievement of most of protein requirements via food intake is not possible during the first weeks after BS. Because of that, the use of protein supplementation is recommended and essential after BS [25, 33]. The amount of protein but also its quality are important to guarantee a correct supply of essential amino acids. In this regard, whey protein supplements are recommended [11]. Some studies show an insufficient intake of protein during the first three months after surgery [21, 35, 38]. Another reason for an inadequate intake is the low adherence of patients to taking protein supplements [35]. In this sense, using new technology like an application could help to increase protein intake and improve adherence [39].

Protein requirements after BS continue to be unknown and general recommendations could be insufficient to cover specific needs. More studies are necessary to determinate the best way to estimate protein requirements of patients undergoing BS, the best protein supplementation to cover them and to develop the optimum strategy to ensure correct adherence of patients to supplementation. In this way, the incidence of complications resulting from insufficient protein intake, such as hypoalbuminemia, loss of muscle mass and increased risk of weight regain, can be reduced.

#### Micronutrients deficiencies

Micronutrients are vital for the proper function of the body, with diet representing the main source. However, micronutrient deficiency is common in PLWO, both before and after surgery [22]. Micronutrient intake is affected after BS not only by the decrease in oral intake, but also by structural and physiological changes in the digestive system [12]. Because of that, post-operative micronutrient supplementation and monitorization is necessary (Table 2) to prevent, detect and treat possible deficiencies and avoid their consequences [11]. Despite the established recommendations, the prevalence of post-surgical micronutrient deficiencies is very high, being more prevalent for some vitamins or minerals detailed below [22].

**Table 2:** Micronutrient requirements after SG and RYGB by the American Association of Clinical Endocrinologists/American College of Endocrinology, The Obesity Society and American Society for Metabolic and Bariatric Surgery (AACE-TOS-ASMBS) [11]

Micronutrient	Doses and recommendations
Thiamine (Vitamin B1)	Minimum: > 12 mg/d Recommendation: 50–100 mg/d From a multivitamin or B-complex supplement
Calcium	1,200 to 1,500 mg/d It should be given in divided doses Calcium carbonate should be taken with meals Calcium citrate can be taken with or without meals
Vitamin D	1,200–1,500 mg/d Dose should be based on circulating vitamin D concentration
Iron	45–60 mg/d of elemental iron From a multivitamin and mineral supplement
Vitamin B12	Orally: 350–1,000 µg/d Parenteral: 1,000 µg monthly
Folate	General recommendation: 400–800 µg/d Women of childbearing age: 800–1,000 µg/d
Vitamin A	5,000–10,000 IU/d Increased dose in patients with history of deficiency
Vitamin K	90–120 µg/d Increased dose in patients with history of deficiency
Vitamin E	15 mg/d Increased dose in patients with history of deficiency
Zinc	RYGB: 8–22 mg/d SG: 8–11 mg/d

##### Iron, vitamin B12 and folic acid

Iron deficiency is one of the most common deficiencies after SG and RYGB affecting around 33% of patients [40] being especially prevalent in menstruating women [33]. This deficiency occurs because of several reasons. On the one hand, intake of iron rich food is dramatically decreased in the first months after BS because of a general low food intake and poor tolerance of iron rich foods. On the other hand, RYGB and SG can induce malabsorption due to the decrease in the absorbing surface and the decrease of hydrochloric acid secretion due to the resection of the greater curvature of the stomach [41]. Furthermore, iron absorption may be compromised by other micronutrient supplements like calcium, thereby requiring a separate intake [33].

Iron supplementation is required after BS. Actual recommendations indicate 18 mg per day of iron from a multivitamin source for males and patients without a history of anemia and 45–60 mg/d for menstruating females [11]. However, these general recommendations should be accompanied by close monitoring to detect and treat possible deficiencies and anemia development.

Vitamin B12 is also a common deficiency after the first-year post BS when the stores of cobalamin decrease [33]. General recommendations for vitamin B12 supplementation differ according to the type of administration; a daily oral intake of 350–1,000 µg or a monthly intramuscular injection of 1,000 µg, independently of the type of surgery are indicated [11]. Some studies show that vitamin B12 deficiency is more prevalent after RYGB than after SG, because in the former of the gastrointestinal surface where vitamin B12 is absorbed is bypassed [42, 43]. Other reasons are poor food intake, lower secretion of intrinsic factor [22] and bacterial overgrowth [43]. The prevalence of vitamin B12 deficiency is high, which could suggest that the recommended supplementation dose is insufficient. Actually, in the studies analyzed it has been observed that the actual supplementation is lower than recommended [42].

Deficiency of folic acid also occurs after BS. In this case, the main reason is a low intake or and inadequate adherence to supplementation because the absorption occurs mainly in the jejunum and it is not affected by BS [33, 42]. In the majority of patients a dose of 400–800 µg/d from a multivitamin source is recommended but special populations such as women of childbearing age should take 800-1,000 µg/d [11].

Iron, vitamin B12 and folic acid deficiency are the main causes of anemia after BS. Data on the prevalence of anemia is heterogeneous in the different studies published due to diverse factors such as different definitions, types of surgery and doses of supplementation [44]. Some studies have estimated an anemia prevalence around 20–30% after BS and the prevalence tends to increase with time, being higher at 2 years postoperatively than after 1 year of BS [45]. A recent meta-analysis concluded that the prevalence of anemia is similar in RYGB and SG [42].

##### Vitamin D and calcium

Vitamin D deficiency continues to be very common after BS. Its prevalence varies between 25% and 73% [12] with a higher prevalence being expected after RYGB than SG. However, a recent meta-analysis showed that there was no difference between both types of surgeries [46]. Vitamin D absorption is decreased after RYGB, mainly because absorption occurs in the duodenum and proximal jejunum that are bypassed in these patients, but also because fat absorption and metabolism is altered after RYGB [22, 47].

Furthermore, hypocalcemia is also very prevalent after BS and may take place following both types of surgery [48]. Several reasons account for calcium deficiency. In addition to a low dietary intake and a poor adherence to supplementation after BS calcium malabsorption is mediated by vitamin D deficiency while the long-term proton-pump inhibitor therapy decreases calcium absorption capacity [11, 47], and the main absorption surface is bypassed [22, 47]. In the last years, prebiotics based on soluble corn fiber have been tried to find ways to increase intestinal calcium absorption after BS [49]. So far, no conclusive results on increased calcium absorption have been obtained, warranting the need of further studies.

Vitamin D and calcium deficiency can lead to bone mineral density loss because of an increase in bone turnover, increasing the risk of fractures [11, 47]. Fracture risk is higher in bariatric patients compared to non-operated patients [12]. In fact, bone health after BS is also affected by other factors such as changes in humoral factors from the pancreas, adipose tissue and the digestive tract (e.g., insulin, leptin, estradiol and ghrelin, among others) [50]. This situation can be aggravated in certain patient groups. Depending on the age of the patient undergoing surgery, the rate of bone loss will increase regardless of gender, while in the postmenopausal women the rate of bone loss is still higher due to the abrupt decrease in estrogens [51]. Thus, the screening of bone mineral density is recommended in postmenopausal women [11].

Calcium and vitamin D supplementation are essential. Actual recommendations are 1,200-1,500 mg/d divided in different doses for calcium with the special consideration that calcium carbonate must be taken with meals. Regarding vitamin D supplementation, there are no specific recommendations for bariatric patients; the dose recommended is at least 3,000 IU/d but depends on serum levels of vitamin D with the aim of maintaining concentrations > 30 ng/mL [11]. Vitamin D circulating levels between 25 and 30 ng/mL seem to be effective in preventing osteoporosis and fracture risk [12]. There are no specific recommendations according to age and sex, whereby continuous screening is necessary to detect deficiencies and adjust supplementation.

##### Liposoluble vitamins

Vitamin A, D, K and E are fat soluble. Their deficiency can occur after BS, being more common after procedures with a malabsorption component than after those with a restrictive component. Therefore, supplementation is recommended [11, 33]. The regular doses may be insufficient for patients with previous history of vitamin A, E or K deficiency. Thus, monitoring is necessary to adjust the doses and prevent deficiencies [11].

##### Vitamin B1 (thiamine)

Thiamine deficiency may occur after SG and RYGB when an inadequate oral intake takes place because the body stores are very limited. This deficiency mainly occurs with persistent vomiting after BS due to a stoma stenosis after RYGB or stomach oedema after SG [33]. Regular supplementation recommendation is 5,000–10,000 IU/d for patients undergoing BS but the supplementation should be higher in patients who present a deficiency. Furthermore, it is necessary to asses iron and copper levels because their deficiency may impair the resolution of the vitamin A deficiency [11].

##### Other micronutrients

In addition to those mentioned above, other deficiencies such as copper, zinc, vitamin B6, magnesium or potassium, among other deficiencies, can appear after BS. The recommended supplementation should be sufficient to avoid their deficiency, but it is essential to carry out an appropriate follow-up and monitoring in order to detect and treat them [11, 33].

The prevalence of micronutrient deficiencies in BS patients is difficult to determine due to the heterogeneity of the studies. In many clinical settings, the supplementation indicated to patients is lower than current recommendations. Adherence of patients to supplementation may also affect the results. In any case, micronutrient deficiency prevalence may be high, and is associated with negative outcomes on the patients’ health. Furthermore, it varies according to the surgical technique used. The RYGB and SG are the most used and studied techniques. However, other BS techniques also involve nutritional deficiencies. In relation to adjustable gastric banding, the prevalence of nutritional deficiencies is usually lower because there is no malabsorptive component. However, deficiencies also occur due to other factors such as a decrease in the amount of food ingested, food intolerance as well as poor dietary habits [25]. However, in techniques with a higher degree of malabsorption due to an increased length of bypassed intestine, such as biliopancreatic diversion, nutritional deficiencies are much more frequent, which is one of the reasons why the use of these techniques has been reduced in recent years [30].

Current micronutrient recommendations may under- and overestimate the real requirements of patients. It is key to insist in applying current recommendations, as well as in carrying out an adequate follow-up of the patients to detect and treat possible deficiencies (Table 3) [11]. Furthermore, currently, today supplementation is based on regular multivitamin supplements, which reduce the rate of deficiencies compared to non-supplemented patients but is insufficient to prevent the high rates of postoperative deficiencies [11, 52]. Because of that, a specialized multivitamin supplement for patients undergoing SG has been developed demonstrating a reduction in prevalence of micronutrient deficiencies as compared to the use of regular supplements [52]. There is no one supplement that fits all patients. Therefore, the use of specialized supplements could help to decrease the rate of deficiencies. More studies are needed to determine with greater accuracy the best supplementation to avoid micronutrient deficiency in patients undergoing BS, considering factors such as sex, age, reproductive performance, presurgical deficiencies and comorbidities that determine the requirements. In the precision nutrition context the aim is to know the adjusted doses for the different patient groups and the best way to proportionate them to prevent future deficiencies and identify the dose required since the initial moment.

**Table 3:** Post-operative monitoring recommendations [11]

Item	SG	RYGB
Control visits (months)	1,3,6,12	1,3,6,12
Weight progress each visit	✓	✓
Lipid evaluation every 6–12 months based on risk and therapy	✓	✓
Bone density (DXA) at 2 years	✓	✓
Vitamin B12 annually	✓	✓
Folic acid, iron studies and 25-OHD3	x	✓
Vitamin A	x	Optional
Copper, zinc and selenium evaluation	x	✓
Thiamine	✓	✓
Adherence to physical activity recommendations	✓	✓

### Follow-up

Multidisciplinary long-term follow-up by obesity specialists, dieticians and nurses specifically trained in bariatric medicine is recommended. Patients who have a good post-operative follow-up adherence reportedly exhibit better results than patients with low adherence [11, 33]. Follow-up is recommended during months 1, 3, 6, 9 and 12 post-surgery. Thereafter, the follow-up becomes annually. It is necessary to evaluate WL progress and remission of comorbidities as well as nutritional status controlling macro- and micronutrient intake and global health state. However, available data shows a low adherence to follow-up appointments, decreasing with the passing of the postoperative months [11]. Thus, it is necessary to develop new strategies to increase adherence.

### Weight regain after BS

After the achieve WL period following BS, which expands for 12–15 months, weight tends to remain stable [7]. However, in a variable percentage of patients who undergo BS, weight regain is observed [5, 53]. The prevalence of weight regain is variable depending on the type of surgery performed, time of postoperative follow-up and criteria for defining weight regain, among other factors [12].

**Fig. 3:**
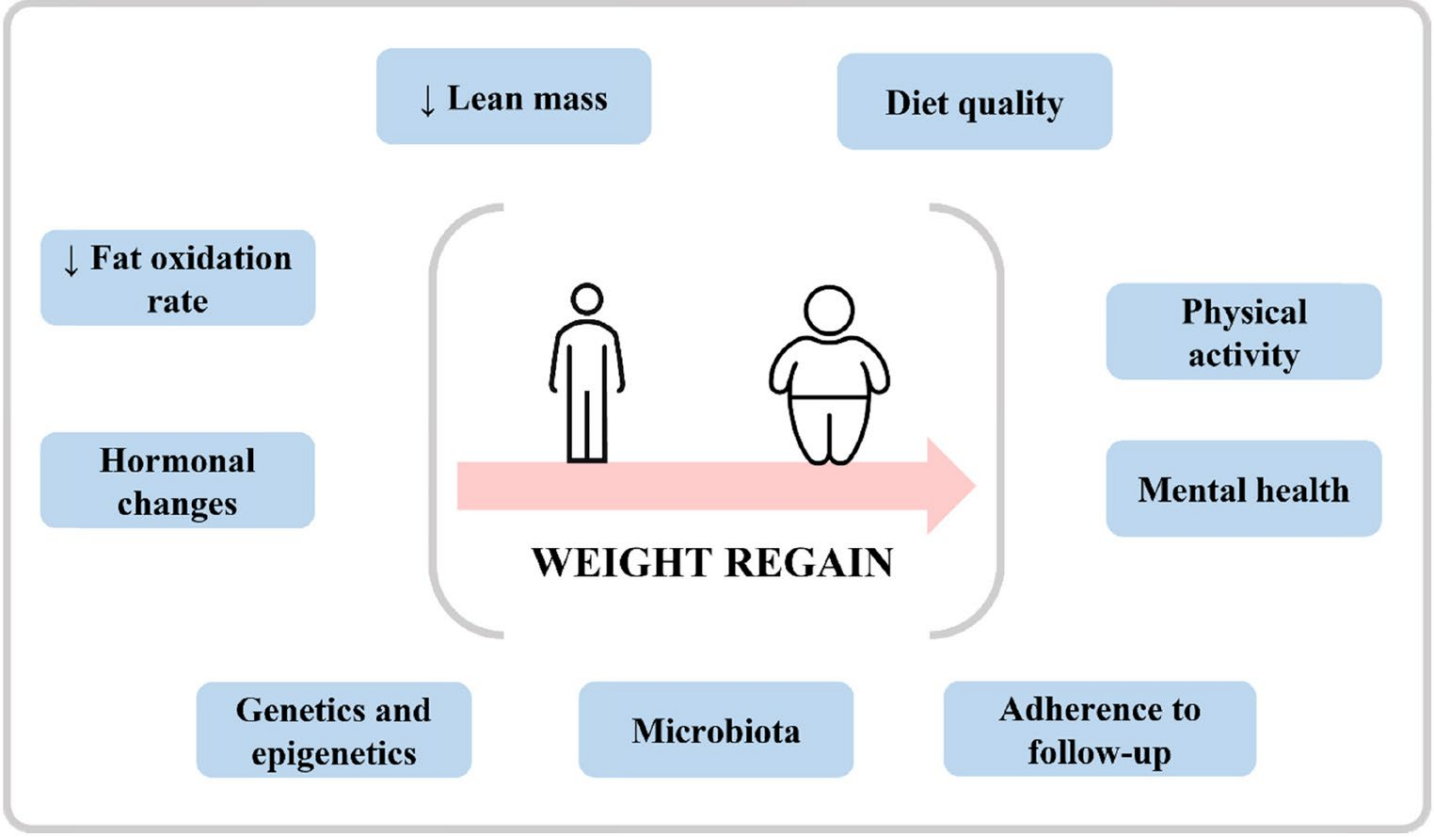
Factors potentially involved in the weight regain that takes place in some patients after BS

The mechanisms that appear to be involved in weight regain are diverse (Fig. 3). On the one hand, a higher loss of lean mass during the first months postoperatively contributes to a reduction in energy expenditure favoring weight gain due to a higher than required energy intake [12, 54]. On the other hand, the hormonal changes produced after BS are being studied in recent years. After BS increasing concentrations of GLP-1, PYY and gastric inhibitory polypeptide (GIP), as well as a decrease in ghrelin levels are observed [55]. However, these changes may not evolve equally in all patients over time. Santo et al. [56] observed that patients with weight regain present lower postprandial levels of GLP-1 and GIP than patients who maintain weight, although the basal concentrations were similar, concluding that these low levels could be involved in the weight regain process. Noteworthy, leptin levels were lower in the weight maintenance group from the very beginning. Therefore, rates of fat oxidation decrease in patients after BS so this could explain the tendency to gain weight after WL in the initial months after surgery [57]. Furthermore, leptin changes can alter the balance between lipolysis and lipogenesis [58–60].

In addition to the factors mentioned above, it is also important to assess adherence to the dietary recommendations as well as the quality of the diet in maintaining WL. A correct intake of protein and fiber, including at least 5 daily servings of fresh fruits and vegetables per day, is important to maintain a balanced diet [11, 33]. Likewise, a diet rich in fish and omega-3 polyunsaturated fatty acids, may increase the Adpn/Lep ratio in relation with a low inflammatory state of the adipose tissue [61, 62]. Ağbaba et al. [63] evaluated the relationship of diet quality with weight change and depression in women who had undergone SG showing that women with better adherence to dietary recommendations and better diet quality present higher WL after a year, concluding that the postoperative follow up is vital to make changes in lifestyle and eating habits to achieve WL and prevent the regain. A recent meta-analysis shown that energy intake, independent to macronutrient proportions, is essential to prevent the weight regain after BS [64]. Diet quality is a protective factor against postoperative weight gain, while time is a risk factor [65]. Therefore, subsequent postoperative follow-up is essential to maintain correct dietary habits and prevent weight regain. Finally, it is important to assess other aspects such as the motivation, the level of physical activity and mental health of patients undergoing BS [12, 57].

### Food preferences and taste after BS

After BS dietary intake is altered mainly by the reduction of the amount of food intake. However, there are other factors to take into account such as food intolerances and changes in taste and food preferences [18, 29]. Food intolerance occurs mainly in the firsts years after BS. In some cases, food intolerance can help to avoid the intake of non-recommended products like sugar due to the dumping syndrome. However, the intake of other foods such as dairy products or meat can also compromise the correct fulfilment of nutritional requirements. In any case, it is believed that the digestive system adapts over time after surgery, improving food tolerance and nutrient absorption [29].

In relation to food preferences, different results between the verbal reports of patients undergoing BS and their eating behavior can be observed [10]. Patients report a decreased appetite for foods rich in sugar and fat [66, 67]. However, several studies have shown that the reduction in caloric intake is due to a decrease in the amount eaten but not in the type of food consumed [68, 69]. Regarding taste after surgery, few studies are available, and their results are inconclusive. It seems that after RYGB a smaller threshold for detection of sweet foods, although it is not known if this conditions the amount of intake [18]. All these changes may be influenced by alterations in the signaling from the gut to the brain. Moreover, changes in the microbiota, in the secretion of digestive hormones such as GLP-1 and ghrelin as well as the decrease in the production of orexigenic neuropeptides in the hypothalamus, may underlie some of these alterations [18]. Nonetheless, more studies are needed to understand these mechanisms in greater detail.

## Other considerations

### Different dietary patterns: vegetarian and vegan diets

Nowadays, dietary patterns are increasingly diverse, which also affects patients who are candidates for BS. The body of knowledge on vegetarian and vegan diets in patients undergoing BS in scarce. The pre-surgical nutritional status of vegetarians compared to omnivorous patients does not exhibit statistically significant differences except for lower levels of plasma glucose and ferritin as well as higher levels of transferrin in the vegetarian group [70]. However, zinc, calcium and iodine concentrations were not analyzed, which would be interesting given that their intake may be decreased in a poorly planned vegetarian diet. Furthermore, it should be noted that this study was carried out in an Israeli population, with different gastronomic habits to those of other countries that could influence the nutritional status of the patients.

The nutritional status of vegetarian patients who are candidates for BS should be studied in detail prior to surgery to detect potential nutritional deficiencies and correct them. Regarding the postoperative period, the planning of vegetarian and vegan diets represents a challenge and should be carried out by a specialized dietician to prevent the appearance of nutritional deficiencies. As mentioned, the intake of macronutrients, especially protein, as well as micronutrients can be compromised, especially during the first postoperative months, leading to nutritional deficiencies. In the case of vegetarian and vegan diets, they may occur more easily due to many of the food sources of these nutrients not being consumed. On the one hand, in relation to protein, it is difficult to reach the protein requirements without consuming animal protein, since foods that are sources of vegetable protein contain a lower amount of them, in addition to the fact that they are more difficult to digest. Because of that, it will be essential to maintain a correct supplementation from whey protein or vegetable proteins such as soy or pea [70]. On the other hand, the dietary intake of some vitamins and minerals can also be expected to be lower in vegan and vegetarian patients, especially iron, vitamin B12, zinc, iodine, calcium, and vitamin D. Therefore, it would be interesting to know whether the standard supplementation proposed for the BS patients is sufficient to avoid nutritional deficiencies in these cases or if it is necessary to increase the doses of all or some of them. Therefore, more studies are needed in this field.

### Sarcopenia: an issue before and after surgery

Sarcopenic obesity (SO) is a clinical and functional condition characterized by the coexistence of obesity, due to an excess fat mass, and sarcopenia [71, 72]. The diagnosis of sarcopenia is based on the presence of at least two of three parameters: low muscle mass, low muscle strength and low physical performance [73]. Although this syndrome is mainly described in elderly people, PLWO of any age can develop sarcopenia due to several factors: adipose tissue-dependent metabolic derangements, chronic non-communicable diseases with negative impact on muscle metabolism, sedentary lifestyle, loss of lean mass during a WL process and weight cycling, among others [71, 72].

Whilst no specific studies have evaluated the prevalence of SO in BS candidates, it is known that WL after BS can be accompanied by a variable degree of loss of lean and bone mass. This, together with the potential post-operative nutritional deficits such as calcium, vitamin D and proteins, can lead to sarcopenia and a decrease in the functional capacity of patients [74]. The prevalence and relevance of SO in BS patients is being studied. Pekar et al. [75] found no cases of sarcopenia in their sample of 19 patients who were analyzed 2 years after BS. However, patients exhibited values close to the diagnosis and in all cases a decrease in lean and bone mass took place, placing the intervened patients at risk of sarcopenia. Buzza et al. [76] have studied the prevalence of sarcopenia in patients who had undergone BS compared to patients with obesity who had not undergone surgery, concluding that low lean mass diagnosis was significantly higher in BS, although it was not followed by a substantial decrease in muscle strength, as seen in the non-significant difference in sarcopenia between BS and non-operated patients. On the other hand, Baad et al. [74] compared the presence of sarcopenia in patients after SG and RYGB concluding that there is no difference in the prevalence of sarcopenia and physical performance between groups, although the preservation of bone and muscle mass was higher in patients undergoing SG.

The interpretation of the results obtained in the available studies is complex due to heterogeneity in criteria and diagnostic methods of SO. However, it is known that patients who have undergone surgery are at risk of developing sarcopenia especially when there is no supervision and follow-up [71]. Regular physical activity is of at most importance to preserve muscle mass. More studies are needed to determine the prevalence of SO among surgical candidates, as well as the impact this could have on their postsurgical evolution. In addition, it is necessary to continue investigating whether the impact of the two types of surgery is the same, and whether this could become a determining factor in the choice of surgery for certain patient profiles such as postmenopausal women, among others.

### Genetics, epigenetics and microbiota

Due to the multifactorial nature of energy homeostasis regulation a huge interindividual variability in response to treatment takes places [4]. In the context of precision nutrition, the influence exerted by genetics and epigenetics factors as well as by the microbiota needs to be considered. Genes associated with obesity development, thermogenesis, adipogenesis and eating behavior reportedly influence WL percentage and maintenance after BS [77]. The study of genetics is aimed at detecting biomarkers useful for predicting the response that PLWO may present to the different therapeutic alternatives in order to select the one that may offer the best results for the patient. The identification of specific single nucleotide polymorphisms that counter more favourable results in response to green dietary patterns or macronutrient distribution has been attempted.

However, so far the cost-benefit relation of these approaches with respect to tangible long-term outcomes needs to be established [78, 79]. Epigenetic changes have also been observed after BS that could be related to WL itself or to other factors such as dietary and hormonal changes or the surgical procedure. Investigations are under way to determine whether methylation of some genes could predict WL after RYGB [77]. These findings will be critical in order to individualize and optimize medical and nutritional treatment of PLWO.

It is known that BS leads to changes in the microbiota. Some studies suggest that changes in the microbiota may not only be a consequence of dietary change and WL after BS but may also contribute to WL itself [10, 77]. More studies are needed to understand the role of changes in the microbiota in the metabolic improvement and WL after BS.

A better understandings of the mechanisms by which all these factors influence treatment response will certainly lead to a more precise and efficient management of PLWO and open up new therapeutic targets that involve nutrition.

### Telemedicine

The progress of new technologies has led to the development of telemedicine in the last years experimenting a further boost due to the COVID-19 pandemic. Telemedicine allows a more frequent and immediate contact between the patient and the healthcare professional. In the context of BS, there are few studies on the use of telemedicine. So far, good acceptance and adherence of patients to this method of communication as well as positive results on the efficacy of telemedicine in achieving changes in physical activity and eating habits of patients have been reported [80]. Moreover, telemedicine also is useful in preoperative education of patients. However, current evidence is low and more studies are needed to develop the best way to integrate telemedicine in the treatment and follow-up of patients undergoing BS to maximize its benefits.

### Sustainability

Over the last few years, there has been growing concern about the need to protect the environment. In this sense, adopting a sustainable diet is a fundamental point to achieve this goal [81]. Because of that, nowadays, dietary guidelines focus not only on the impact of foods and nutrients on health, but also on the protection of the environment and overall sustainability of diets [82]. Therefore, in the context of dietary recommendations following BS, sustainability must be an additional point to consider. Diets should be made up, whenever possible, with local or kilometer 0 foods, as well as adapted to contain seasonal foods. In this way, it will be possible to contribute to protection of biodiversity as well as ecosystems and contribute to a more healthy life of the present and future generations [81].

## Conclusion

BS is currently the most effective treatment option for severe obesity. However, eating is dramatically affected after BS. Therefore, it is essential to follow dietary recommendations and supplementation of protein, minerals and vitamins. Despite this, nutritional deficiency rates remain high in these patients. Among the factors involved not only poor patient adherence to recommendations stand out, but also a lack of knowledge of the real nutritional requirements of the patients undergoing BS needs to be contemplated. Because of that, the actual general recommendations may be insufficient. It is necessary to develop methods for the determination of the nutritional requirements, specifically protein and micronutrients, of patients undergoing BS, according to their individual characteristics such as age, sex and nutritional status, among others, prior to surgery. Likewise, it is necessary to continue studying to determine the best way to cover these requirements, through the development of micronutrient supplementation that better adapts to the requirements of this population. All this will allow us to evolve from the current general recommendations to specific tailor-made recommendations applying precision nutrition. Moreover, the contribution of genetic and epigenetic factors as well as of the microbiota, among others, will need to be incorporated in a precision nutrition approach. To date, knowledge in these fields is limited. However, efforts to understand the involvement of these factors at the same time as warranting sustainability and a low carbon footprint should be pursued.

## Abbreviations

AACE: American Association of Clinical Endocrinologists
ASMBS: American Society for Metabolic and Bariatric Surgery
BMI: Body mass index
BS: Bariatric surgery
ESPEN: European Society for Clinical Nutrition and Metabolism
GLP-1: Glucagon-like peptide-1
OXM: Oxyntomodulin
PLWO: People living with obesity
PPY: Peptide YY
RYGB: Roux-en-Y gastric bypass
SG: Sleeve gastrectomy
SO: Sarcopenic obesity
TOS: The Obesity Society
WL: Weight loss
